# Effect of emulsifying stability of myofibrillar protein on the gel properties of emulsified surimi gel

**DOI:** 10.1002/fsn3.663

**Published:** 2018-05-08

**Authors:** Yuanpei Gao, Hideto Fukushima, Shanggui Deng, Ru Jia, Kazufumi Osako, Emiko Okazaki

**Affiliations:** ^1^ Department of Food Science and Technology Tokyo University of Marine Science and Technology Tokyo Japan; ^2^ Department of Marine Science and Resources Nihon University Fujisawa Japan; ^3^ Department of Food Science and Technology Zhanjiang Ocean University Zhoushan China

**Keywords:** emulsification, fish oil, gel property, microstructure observation, surimi gel

## Abstract

Several kinds of emulsified surimi gels were prepared from different quality levels of Alaska Pollack surimi, and the relationship between the emulsifying stability (ES) of myofibrillar protein and the properties of the emulsified surimi gels was investigated. Fish oil emulsified into surimi gels enhanced the breaking strength, but this was decreased by denaturation of the surimi protein, and the rate of enhanced gel‐forming ability with emulsification decreased with decreasing ES. Expressible drip also decreased with emulsification; however, increasing amounts of lipid in the expressible drip were separated out from the gel upon protein denaturation of the source surimi. Scanning electron microscopy revealed that the shape of fish oil particles became irregular and some voids caused by oil leakage were observed with increasing storage period of source surimi. The results suggested that improvement in gel properties of the emulsified surimi gels was correlated with ES as well as the level of protein denaturation.

## 1 INTRODUCTION

Recently, there has been increasing interest in the incorporation of polyunsaturated fatty acids (PUFAs), such as eicosapentaenoic acid (EPA) and docosahexaenoic acid (DHA), to foods because of their many health benefits. The benefits of fish oils can largely be attributed to the high PUFA content (Kris‐Etherton, 2002; Nair, Leitch, Falconer, & Garg, 1997). The consumption of DHA and EPA reduces the risk of hypertension and Alzheimer's disease (Tully et al., 2003), and prevents certain cardiac arrhythmias (Garg, Wood, Singh, & Moughan, 2006). Moreover, a previous study confirmed the benefits of EPA and DHA supplementation during pregnancy on fetal brain and retina development (Ramakrishnan et al., 2010). As noted above, EPA and DHA are among the few functional ingredients that have been clinically confirmed to be effective for human health. To enhance the functionality and economic value of foods, researchers have successfully fortified many kinds of foods such as bread, ice cream, milk, and others with fish oil (Kolanowski & Berger, 1999; Newton & Snyder, 1997). Moreover, these functional foods have recently been marketed, and further application of fish oil to the food industry is expected. On the other hand, it is necessary to minimize changes in the physical properties of foods supplemented with fish oil as well as prevent the generation of fishy odors, thus, a number of issues remain to be solved.

Surimi‐based foods are widely accepted and enjoyed throughout the world. Surimi is a mechanically deboned, washed (bleached), and stabilized fish meat (Okazaki & Kimura, 2013) containing myofibrillar (Mf) protein as the main protein. During processing, Mf protein is solubilized using salt to form a viscous state, which can then be mixed with a range of additives and processed into various forms. Therefore, surimi is a convenient intermediate product used in the preparation of a number of ready‐to‐eat seafood such as kamaboko, fish sausage, imitation crab legs, and shrimp products (Claus, Colby, & Flick, 1994). Recently, surimi‐based foods enriched with EPA and DHA have demonstrated potential wide applicability.

In general, it had been considered difficult to produce surimi containing high amounts of oil, as fish protein and oil are not compatible and are thus difficult to mix uniformly and stably, leading to deterioration in product quality, for example, gel‐forming ability. On the other hand, Ikeuchi and Simizu (1955) suggested that appropriate amounts of added oil to fish meat might be enclosed safely within the fish meat protein network in a stable oil–water emulsion state. In previous research, Okazaki, Noda, Fukushima, and Fukuda (2006) and Okazaki, Yamashita, and Omura (2002) reported that in the preparation method of emulsified surimi with fish oil, the physical properties of surimi gel were improved with decreasing oil particles size under vigorous mixing conditions. Debusca, Tahergorabi, Beamer, Partington, and Jaczynski (2013) reported that the fortification of surimi seafood with either dietary fiber or omega‐3‐rich oils alone or in combination improved the rheological and textural characteristics of surimi gels. On the other hand, Benjakul, Visessanguan, and Kwalumtharn (2004) and Shi et al. (2014) reported that the addition of vegetable oil to surimi gels enhanced the white color but reduced the gel‐forming ability of surimi gels with the same moisture content. Fukushima, Okazaki, Noda, and Fukuda (2007) reported that the emulsification of fish oil into surimi gel enhanced the whiteness, gel‐forming ability, and water holding capacity. In this type of emulsification system, Mf protein is suggested to play an important role in stabilizing the lipid particles, which is related to the emulsifying stability, and may contribute certain positive or negative effects to the final product; however, detailed information on this point is not yet available. To date, few studies have focused on the relationship between the emulsifying stability of Mf protein and the gel properties of surimi/fish oil emulsion systems. Therefore, this study aimed to clarify the relationship between the emulsifying stability of Mf protein and the properties of emulsified surimi gels using different quality levels of surimi.

## 2 MATERIALS AND METHODS

### 2.1 Materials

#### 2.1.1 Frozen surimi

Alaska Pollock (*Theragra chalcogramma*) surimi (FA grade) was produced by Pacific Seafoods Corp., Portland, OR. The surimi was cut into small blocks (about 500 g), vacuum packed, and stored at −30°C until use. The surimi contained 75.5% moisture, 17.0% crude protein, 0.4% crude lipid, and 7.1% others.

#### 2.1.2 Preparation of different quality levels of surimi

**Figure 1:**
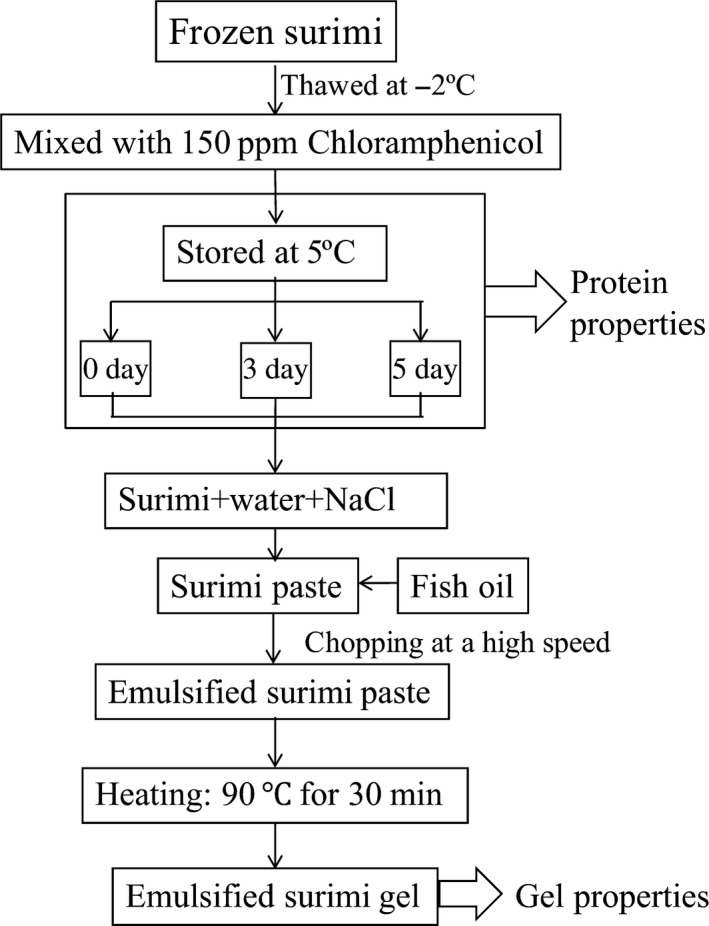
Flowsheet of the experiment design

The flowsheet of experiment design is shown in Figure 1. Frozen surimi was thawed at −2°C and mixed with 150 ppm chloramphenicol using a food processor to prevent spoilage. The processed surimi was vacuum packed and stored at 5°C for 0, 3, and 5 days to prepare the different levels of quality.

#### 2.1.3 Fish oil

Refined fish oil, DHA‐27W, was prepared from tuna and bonito by Maruha Nichiro Corp., Tokyo, Japan, and stored at −30°C until use. The fish oil contained more than 99% triglyceride, and 0.6% tocopherol as an antioxidant. DHA and EPA contents were 24.4% and 4.4%, respectively.

### 2.2 Solubility of surimi protein

A 2 g portion of surimi was mixed with 40 ml of 0.6 mol/L NaCl and homogenized at 11,000 × *g* with a homogenizer (PT 10‐35 GT; Kinematica, Lucerne, Switzerland) for 30 s. The homogenate was centrifuged at 39,000 × *g* for 20 min at 4°C. The supernatant was collected and subjected to determination of protein concentration by the method of Lowry, Rosebrough, Farr, and Randall (1951). Solubility was expressed as the ratio of protein content in the supernatant of the stored samples to that of the sample without storage.

### 2.3 Preparation of surimi myofibrillar protein (Mf) suspension

The surimi Mf suspension was prepared by the method Hashimoto and Arai (1979) with some modifications. A 2.5 g portion of surimi was homogenized (PT 10‐35 GT; Kinematica) in 20 ml buffer solution (0.16 mol/L KCl‐40 mmol/L Tris–HCl, pH 7.5), followed by centrifugation for 7 min (3,000 × *g*, 4°C). The supernatant was discarded, and the precipitate was collected. This procedure was repeated four more times. After filtering the suspension with gauze at last time, the protein concentration was measured by method of Lowry et al. (1951) and adjusted to 2.5 mg/ml using the same buffer solution.

### 2.4 Ca^2+^‐ATPase activity and emulsifying stability index (ESI)

The Ca^2+^‐ATPase activity of surimi Mf was measured using the method of Kato, Uchiyama, Tsukamoto, and Arai (1977) with some modifications. A 200 μl aliquot of Mf suspension was mixed with a solution (100 μl of 0.5 mol/L Tris–Maleic acid buffer, 100 μl of 0.1 mol/L CaCl_2_ solution, 440 μl of 2 mol/L KCl solution and 1,060 μl of IEW) at 25°C for 10 min, then 100 μl of 20 mmol/L ATP solution was added to start the reaction, and after 15 min, 100 μl of 15% PCA was added to stop the reaction. After centrifugation (4,000 × *g*, 10 min), 1 ml of the supernatant was reacted with the following reagent (0.5 ml of sulfuric‐molybdic acid, 250 μl of Elon solution and 1.25 ml of IEW) for 45 min and absorbance was measured at 640 nm using a spectrophotometer (Model UV‐1800; Shimadzu Corp., Japan). A standard curve was prepared using potassium phosphate solution.

Ca^2+^‐ATPase activity (μmol/mg protein/min) = [(*A*−*B*)/*t*]/*P*

where *A* is the content of phosphoric acid (μmol), *B* is the content of phosphoric acid in the blank group (μmol), *t* is reaction time, and *P* is protein content (mg).

The emulsifying properties of Mf were determined according to the method of Pearce and Kinsella (1978). To prepare emulsions, 2.0 ml of fish oil and 8.0 ml of protein solution in 0.1 mol/L phosphate buffer (1 mg/ml protein) were homogenized at 15,000 rpm for 1 min (PT10‐35GT; Kinematica). A sample of freshly prepared emulsion (50 μl) was taken 0.5 cm from the bottom of the tube and dispersed into 5 ml of 0.1% sodium dodecyl sulfate (SDS) solution. The absorbance was measured at 500 nm against 0.1% SDS solution as the blank. Emulsions were left undisturbed for 10 min at 20°C and then 50 μl of emulsion was taken 0.5 cm from the bottom of the tube and dispersed into 5 ml of 0.1% SDS solution. The absorbance was measured at 500 nm. Emulsifying stability index, expressed as ESI (%), was defined as:

ESI = (A10/A0) × 100

where A10 and A0 represent the absorbance at 500 nm after 10 min and time zero after homogenization, respectively.

### 2.5 Preparation of emulsified surimi paste and heat‐induced gels

The stored source surimi was chopped in a universal food processor (Model UMC 5E; Stephan Machinery, Hameln, Germany) at 1,500 rpm for 1 min. Surimi paste was obtained after mixing with cold IEW and NaCl. The ingredients of the control and emulsified groups are shown in Table 1. For type A gel, fish oil was added into the surimi paste instead of water, and it had the same protein content as the control group. Consequently, the ratio of protein/moisture was higher than the control due to the addition of fish oil. For type B gel, the ratio of protein/moisture was the same as the control group. After adding the fish oil, emulsified surimi paste was prepared by high‐speed mixing, using a stepwise increase in speed from 300 to 3,000 rpm as follows: 300 rpm for 60 s; 600, 900, 1,200, 1,500 rpm for 30 s; 1,800, 2,100 rpm for 15 s and 2,400, 2,700, 3,000 rpm for 10 s, respectively (Okazaki et al., 2002). The above operation was repeated twice. All steps were performed at temperatures below 10°C under vacuum. The prepared surimi paste was packed into polyvinylidene chloride casing tubes 23 mm in diameter, and heat‐induced gel was obtained by two methods: direct heating (90°C for 30 min) and two‐step heating (30°C for 60 min, then 90°C for 30 min). After heating, the samples were cooled in ice water for more than 30 min.

**Table 1:** Ingredients of surimi gels (g)

	Surimi	IEW	NaCl	Fish oil	Total
Control	544	240	16	0	800
Emulsified A^a^	544	160	16	80	800
Emulsified B^b^	544	240	16	80	880

a Same protein concentration with control, but different protein/moisture ratio.

b Same protein/moisture ratio with control.

### 2.6 Proximate composition

Moisture content was determined according to AOAC (1995). Total lipid content was determined according to the method of Folch, Lees, and Sloane‐Stanley (1957). Total nitrogen content was determined by the Kjeldahl method (Bradstreet, 1954), and a nitrogen conversion factor of 6.25 was used to calculate the protein content. All proximate analyses are expressed as mean values of at least three replicates.

### 2.7 Physical properties

Breaking strength (g) and breaking strain (mm) of heat‐induced surimi gels were measured by puncture‐test using a rheometer (Rheometer NR M‐2002J; Fudoh Industry Co., Ltd., Tokyo). Measurements were conducted using a 5 mm spherical plunger at a speed of 6 cm/min. All measurements were repeated six times.

### 2.8 Expressible moisture and others

Expressible moisture and others were measured by the method of Rawdkuen and Benjakul (2008) with some modifications. Cylindrical gel (moisture content was X) samples were cut to a weight of approximate 1.0 g, weighed (W1) and placed between two types of filter paper (No. 2 and No. 4A, Advantec, Inc., Tokyo, Japan), No. 2 on the outer side and No. 4A on the inner side, at the bottom, and the top of sample. A constant force (20N) was placed on top of the sample for 20 s using a rheometer (RE‐3350; Yamaden Ltd., Tokyo, Japan), and then the sample was removed from the papers and weighed again (W2). The pressed gel was dried at 105°C for 24 hr and weighed again (W3).

Total expressible drip (TED) (%) = (W1−W2)/W1 × 100

Expressible moisture (EM) (%) = [X × W1 − (W2 − W3)]/W1 × 100

Others = TED − EM (Main component in others is lipid.)

### 2.9 Rheological properties of surimi paste

The rheological properties of surimi paste were measured using a HAAKE MARS 60 Rheometer (Thermo Fisher Scientific Inc, Yokohama, Japan) equipped with a 35 mm parallel plate geometry, and the sample table was covered with paraffin oil to prevent dehydration. Following the method of Fukushima, Satoh, Nakaya, Ishizaki, and Watabe (2003), temperature sweep analysis to measure the changes in rheological parameters, including elastic modulus (G′) and viscous modulus (G″), during heating was performed at a constant frequency of 1 Hz and an amplitude strain of 2%, which was tithing the linear viscoelastic region. The temperature sweep increased from 10–90°C at a heating rate of 2°C/min.

### 2.10 Scanning electron microscopy (SEM)

Gel samples 2 mm in thickness were fixed in 4% paraformaldehyde phosphate buffer solution overnight and immersed in 1% osmium tetroxide solution for over 24 hr. Then, samples were dehydrated in a graded series of 50–70–80–90 and 100% ethanol, at 30 min in each concentration (Rawdkuen & Benjakul, 2008). Finally, dried samples were mounted on a bronze stub and sputter‐coated with gold (E‐1030; Ion Sputter, Hitachi, Ltd., Tokyo, Japan), and then observed by SEM at an acceleration voltage of 10 kV (S‐4000B; Scanning Electron Microscope, Hitachi, Ltd., Tokyo, Japan).

### 2.11 Statistical analysis

The data were expressed as means ± standard deviations. Differences between variables were evaluated using Duncan's multiple range test. Analysis was performed using SPSS Statistics software (SPSS Inc., Chicago, IL).

## 3 RESULTS AND DISCUSSION

### 3.1 Proximate composition and protein properties

The proximate composition of surimi gels is shown in Table 2. There was no significant difference (*p* > .05) in protein concentration between the control and emulsified A gels, as fish oil was added in place of water. In the case of emulsified B gel, the protein concentration was lower than the other groups, but the protein/moisture ratio was the same as the control.

**Table 2:** Proximate composition of surimi gels (g/100 g)

	Moisture	Crude protein	Crude lipid	Others	Protein/Moisture
Control	80.4 ± 0.9^a^	11.1 ± 0.4^a^	0.5 ± 0.02^c^	7.5 ± 0.4^a^	0.14 ± 0.1^b^
Emulsified A	70.3 ± 1.1^c^	11.6 ± 0.3^a^	10.3 ± 0.8^a^	7.8 ± 0.3^a^	0.17 ± 0.1^a^
Emulsified B	72.9 ± 0.2^b^	10.1 ± 0.3^b^	9.0 ± 0.3^b^	6.8 ± 0.1^b^	0.14 ± 0.1^b^

Values are given as means ± *SD*.

Different superscripts in the same column indicate significant differences (*p* < .05).

**Figure 2:**
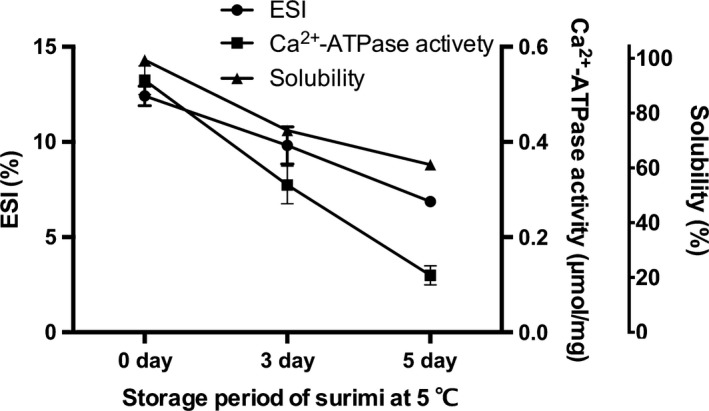
ES, Ca^2+^‐ATPase activity, and solubility of Mf protein at different storage periods after thawing. Bars represent the standard deviations

Figure 2 shows the changes in emulsifying stability index (ESI), Ca^2+^‐ATPase activity and solubility of Mf protein, as the most important resource protein in surimi, during storage after thawing. The Ca^2+^‐ATPase activity, which is a primary indicator of protein denaturation during storage and has been widely used as an indicator of fish or surimi protein denaturation (Donald & Lanier, 1994), decreased with the deterioration of surimi quality by storage at 5°C. Emulsifying activity is dictated by protein–protein and protein–lipid interactions, and emulsifying stability is affected by a broad range of factors that influence both the dispersed and the continuous phases (Ramırez‐Suárez & Xiong, 2003). The emulsifying stability result showed the same tendency as the change in Ca^2+^‐ATPase activity and solubility, significantly (*p* < .05) decreasing in the surimi in a storage period‐dependent manner. Xia, Kong, Liu, and Liu (2009) and Xia, Kong, Xiong, and Ren (2010) also reported a similar result while reducing the protein quality and suggested that the lowered ES might be due to a lower content of flexible peptides able to migrate to the oil–water interface; thus, the decrease in ES is related to Mf protein denaturation.

### 3.2 Physical properties

**Figure 3:**
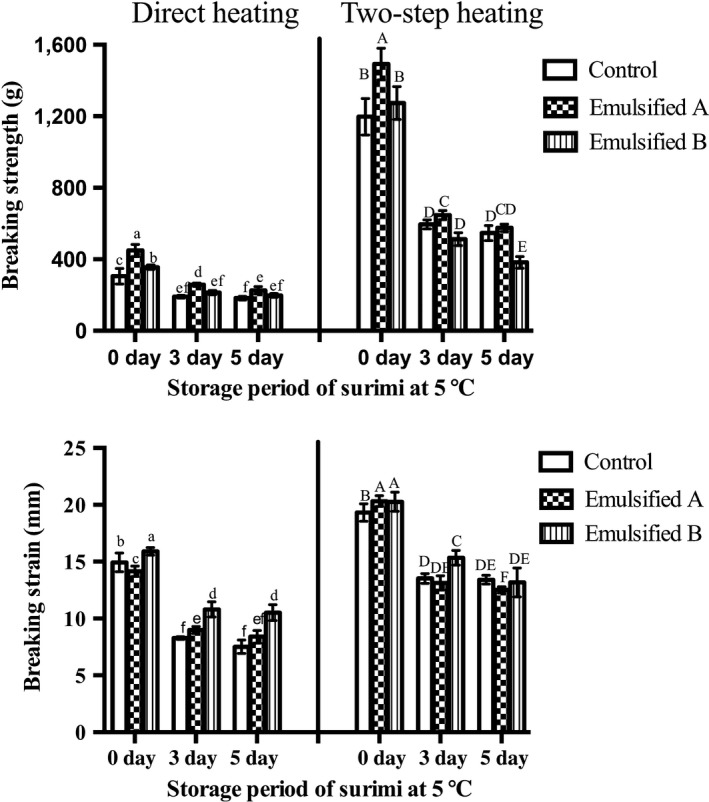
Breaking strength and breaking strain of surimi gels prepared from surimi exposed to different storage periods after thawing. Bars represent the standard deviations and different letters at the same heating conditions indicate significant differences (*p* < .05)

The results for breaking strength and breaking strain are shown in Figure 3. The gel‐forming ability of the gels subjected to two‐step heating was significantly higher compared to direct heating due to the suwari procedure (Kimura, Tahergorabi, Kaneniwa, Sakai, & Tahergorabi, 1991) in both the control and emulsified groups. Both breaking strength and breaking strain decreased with increasing storage period of the source surimi because of protein denaturation, thereby preventing the formation of a tight protein matrix during heat‐induction. For surimi stored for 0 days, the addition of fish oil significantly enhanced the breaking strength of both A and B types of emulsified surimi gels (*p* < .05). In reference to Fukushima et al. (2007) and Okazaki et al. (2006), even though type A gel had the same protein concentration as the control, the added lipid could not be dissolved in the protein‐moisture system. Therefore, type B emulsification, which contained the same protein/moisture ratio as the control, was produced. The result confirmed that the emulsification of fish oil had a positive effect on the physical properties of emulsified surimi gels, even in the case of B type, which contained a lower protein content than the control. Park, Kelleher, McClements, and Decker (2004) also reported that surimi that contains beneficial omega‐3 fatty acids could be developed with good oxidative stability and gel strength. In the case of 3 and 5 days storage periods of the source surimi, the breaking strength of the emulsified group was also enhanced by the addition of fish oil, but not the B group emulsification upon two‐step heating. Table 3 shows the increasing rate of breaking strength of the emulsified gel compared to the control. The effect of fish oil emulsification on the breaking strength of surimi gels was reduced with increasing storage period of the source surimi. Some gels, such as type B, made with surimi stored for 3 or 5 days and subjected to two‐step heating showed a decline in improvement rate to −13.92% and −30.01%, respectively, resulting in an overall negative effect following emulsification. This phenomenon might be attributable to the decrease in the emulsifying stability of Mf protein. Therefore, we hypothesized that Mf protein with the highest ES contributed to stable protein–lipid interactions in the surimi gel. Moreover, the stable protein membrane surrounding the emulsified oil particle would have positive effects on the gel structure stability and gel breaking strength compared to surimi gel without added fish oil. Meanwhile, decreases in ES might damage the gel structure, resulting in a decline of physical properties compared to the control. Breaking strain showed almost the same tendency as breaking strength.

**Table 3:** Increasing rate^a^ of breaking strength of emulsified gels compared to the control gel (%)

Storage period of source surimi	Emulsified A		Emulsified B	
	Direct heating	Two‐step heating	Direct heating	Two‐step heating
0 days	47.14	24.62	16.41	6.37
3 days	35.20	8.80	12.23	−13.92
5 days	29.31	5.35	8.56	−30.01

a (Breaking strength of emulsified group − Breaking strength of control)/Breaking strength of control.

### 3.3 Expressible moisture and others

**Figure 4:**
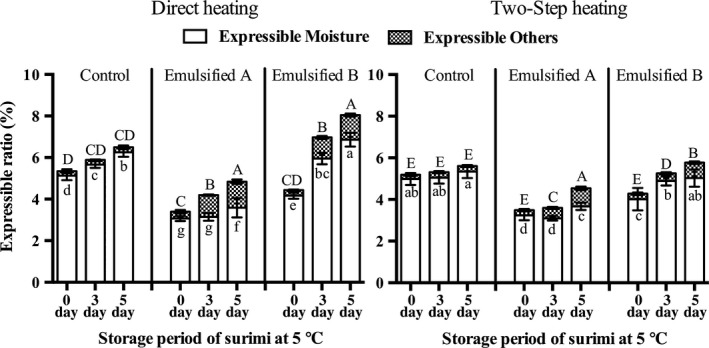
Expressible moisture and lipid of surimi gels prepared from surimi exposed to different storage periods after thawing. Bars represent the standard deviations and different letters within the same heating conditions indicate significant differences (*p* < .05)

Expressible water can indicate the strength of the gel network; the higher the cross‐link density of the gel, the lower the expressible liquid (Mao & Wu, 2007). The result of expressible moisture and others of surimi gels is shown in Figure 4. Total expressible liquid (moisture and others including lipid) in emulsified groups (both A and B) was significantly lower (*p* < .05) than that in the control group at 0 day storage of the source surimi. Normally, in Mf meat products, the lipid is minced into small particles, which is stabilized by membrane‐coating with the salt‐soluble Mf protein (Gordon, Barbut, & Schmidt, 1992). Then, these lipid particles are further stabilized by the protein matrix during the gelation process (Xiong, 2007). This result indicated that the positive effect of emulsification on the stabilization of water and lipid in surimi gels also resulted in the suppression of released liquid. In contrast to the present study, Shi et al. (2014) reported higher expressible water in emulsified surimi gels, due to a decrease in protein concentration, resulting in a lower matrix density. However, Fukushima et al. (2007) reported that expressible drip decreased when fish oil was added into surimi gels with sufficient emulsification at the same protein/moisture ratio, and further decreases in expressible drip were observed with increasing oil content, even though the lower protein content in the B type gel.

On the other hand, total expressible liquid increased with increasing storage period of the source surimi after thawing because Mf protein denaturation progressed gradually, and a weaker protein matrix formed during the heating process, as a consequence, there was greater expressible liquid observed. The degree of change in the amount of released liquid from gels in relation to the storage period of the source surimi differed among each group and was higher in the emulsified groups than the control group. In the emulsified groups, not only expressible moisture but also the other components in the expressible liquid (main component was lipid) significantly increased (*p* < .05) with the storage period of the source surimi and was suspected to be due to decreased ES. Therefore, the protein membrane could not tightly attach to the surface of oil particles to form a stable emulsion system, resulting in the release of lipid by external forces.

### 3.4 Rheological properties

**Figure 5:**
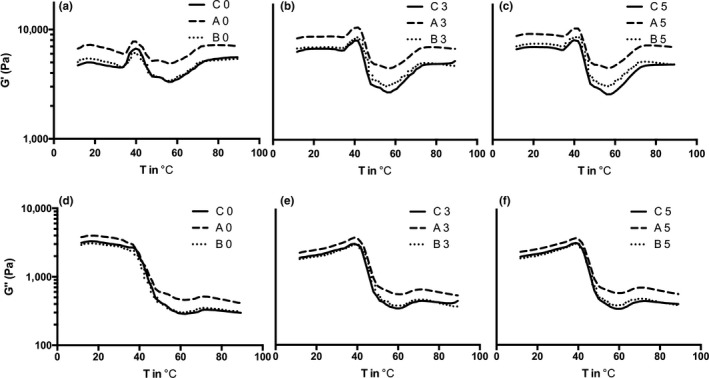
Rheological properties of surimi paste prepared with surimi exposed to different storage periods after thawing. Letter a–c and d–f reveal elastic modulus (G′) and viscous modulus (G″), respectively. C, Control group; A, Emulsified A group; B, Emulsified B group; Number, Storage period of source surimi after thawing

Dynamic rheological measurement can determine changes in the rheological properties of samples serially and nondestructively using variables such as time and temperature. Surimi gelation occurs in two steps: (1) initiation of gelation is characterized by the unfolding of protein molecules, and (2) protein aggregation resulting in gel formation occurs via disulfide bonds as well as hydrogen, electrostatic, and hydrophobic interactions (Egelandsdal, Martinsen, & Autio, 1995; Hamann & MacDonald, 1992). Figure 5 shows that the storage modulus (G′) increased at low temperatures (around 20°C) and this stage was related to the formation of the surimi gel 3D structure (Lefèvre, Fauconneau, Ouali, & Culioli, 1998). A second peak appeared at a temperature around 36°C and reached the highest point at 40°C, termed the gelation point (Reed & Park, 2011); this increase was likely due to the breakage of hydrogen bonds that maintained the folded protein structure, leading to protein unfolding (Liu, Zhao, Xiong, Xie, & Liu, 2007).

From Figure 5a–c), the elastic modulus (G′) of the emulsified surimi paste was higher than the control at 10°C, and resulted from the decrease in fluidity of the emulsified meat paste. G′ of surimi paste prepared with preserved surimi (3 and 5 days) had a higher value than that made with fresh (0 day) surimi at a temperature below 40°C, which was attributed to the denaturation and decreased salt solubility of Mf protein during storage. Thus, the surimi paste could not form a viscous state, leading to a higher elastic modulus under lower temperatures. Additionally, the high activity of endogenous proteolytic enzymes was able to degrade myosin and resulted in a breakdown of the gel structure at temperatures around 60°C. Subsequently, G′ increased gradually until the temperature reached 90°C.

Figure 5d–f shows changes in G″ during the heating process for surimi not subjected to storage (Figure 5d); G″ decreased from 20°C due to the formation of an elastic gel. After storage (Figure 5e,f), on the other hand, G″ decreased from 40°C, possibly due to protein denaturation during surimi storage, resulting in a loss of elasticity.

During the heating process, type A showed higher G′ and G″ than the control and type B, which was attributed to the higher protein concentration in the gel (as described in Section 3.2), and type B showed a similar tendency as the control. On the other hand, the gelation point (the peak appeared around 40°C in Figure 5a–c) was not shifted by addition of fish oil. These results demonstrated that the addition of fish oil itself did not greatly affect the heating‐gelation process of surimi gels, however, G′ and G″ were strongly affected by the storage of source surimi because protein properties were seriously damaged as descripted in the Section 3.1.

### 3.5 Microstructure observation

**Figure 6:**
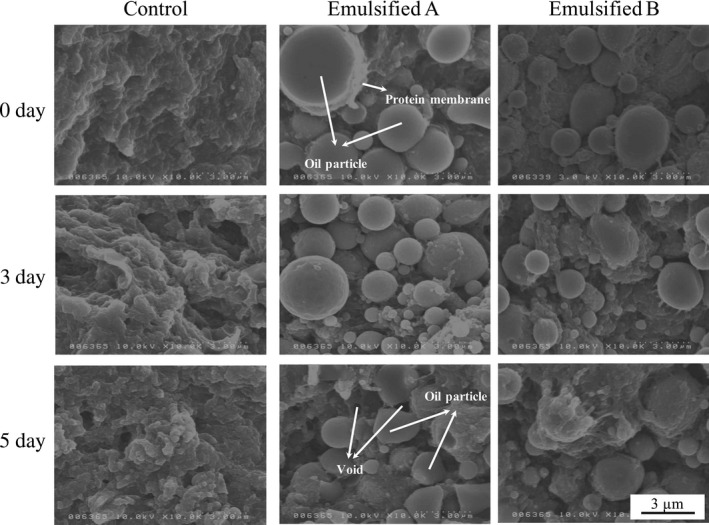
SEM observation of surimi gels prepared from surimi exposed to different storage periods after thawing. With 10 k magnification

Figure 6 shows the results of SEM observation of several heat‐induced gels. Gels prepared from surimi stored for 0d could form spherical and regular oil particles surrounded by a protein membrane. Okazaki et al. (2002) prepared emulsified surimi gels with fish oil using a vacuum chopper at high chopping speed. They found that lipid particles could be dispersed into the final gel at a diameter less than 10 μm and the addition of lipid enhanced the breaking strength; however, they did not focus on the emulsifying stability of Mf protein. Wu, Xiong, and Chen (2011) suggested that the protein membrane serves as a barrier; therefore, the protein membrane and its matrix would provide an additional restriction to the mobility of small oil droplets, that is, critical to their thermal stability. In the present study, oil particles became irregular with decreasing ES. Furthermore, some voids occurred following the release of lipid in the emulsified gels prepared with surimi stored for 5 days, as shown in Figure 6. Tolasa, Lee, and Cakli (2010) reported that omega‐3 fatty acids were not distributed uniformly in surimi gels prepared with low‐grade surimi, which is similar to our present result. However, they observed oil particles using an optical microscope and did not focus on the emulsifying stability of Mf protein. In light of our SEM observation, the lipid and protein structure of surimi gels was altered by the decrease in ES, resulting in diminished gel properties.

## 4 CONCLUSION

The present study indicated that the emulsification of fish oil into surimi gels not only increased its functionality for human health, but also promoted gel properties such as water holding capacity and gel‐forming ability. In particular, improvement of the properties of the emulsified surimi gels was associated with the emulsifying stability of Mf protein in the surimi. From the result, the greater the stability of the protein membrane surrounding the oil particles, the superior the obtained gel properties, and it was hypothesized that the protein film or membrane on the surface of oil particles influences the overall properties of Mf protein gels as well as the quality of the final products. These results suggest that the improving effect on gel properties by emulsification of surimi is related to the formation of a protein membrane surrounding the oil particles emulsified within the surimi gel.

## CONFLICT OF INTEREST

The authors declare that they have no conflict of interest.

## ETHICAL STATEMENT

This article does not contain any studies with human participants or animals performed by any of the authors.
